# Oxidation Enhances Human Serum Albumin Thermal Stability and Changes the Routes of Amyloid Fibril Formation

**DOI:** 10.1371/journal.pone.0084552

**Published:** 2014-01-08

**Authors:** Giuseppe Sancataldo, Valeria Vetri, Vito Foderà, Gianluca Di Cara, Valeria Militello, Maurizio Leone

**Affiliations:** 1 Dipartimento di Fisica e Chimica, Universita' di Palermo, Palermo, Italy; 2 Consiglio Nazionale delle Ricerche-IBF u.o. Palermo, Italy; 3 Department of Drug Design and Pharmacology, University of Copenhagen, Copenhagen, Denmark; 4 Sector of Biological and Soft Systems, Department of Physics, Cavendish Laboratory, University of Cambridge, Cambridge, United Kingdom; 5 Centro di Oncobiologia Sperimentale, Palermo, Italy; National Institute for Medical Research, Medical Research Council, London, United Kingdom

## Abstract

Oxidative damages are linked to several aging-related diseases and are among the chemical pathways determining protein degradation. Specifically, interplay of oxidative stress and protein aggregation is recognized to have a link to the loss of cellular function in pathologies like Alzheimer's and Parkinson's diseases. Interaction between protein and reactive oxygen species may indeed induce small changes in protein structure and lead to the inhibition/modification of protein aggregation process, potentially determining the formation of species with different inherent toxicity. Understanding the temperate relationship between these events can be of utmost importance in unraveling the molecular basis of neurodegeneration. In this work, we investigated the effect of hydrogen peroxide oxidation on Human Serum Albumin (HSA) structure, thermal stability and aggregation properties. In the selected conditions, HSA forms fibrillar aggregates, while the oxidized protein undergoes aggregation via new routes involving, in different extents, specific domains of the molecule. Minute variations due to oxidation of single residues affect HSA tertiary structure leading to protein compaction, increased thermal stability, and reduced association propensity.

## Introduction

Protein molecules are fundamental for cell activities. In some instances both intracellular and extracellular proteins are subjected to a variety of spontaneous non enzymatic changes, which affect their structure, function and stability [1]–[4]. Among these, oxidative damages lead to specific modifications of proteins frequently observed in several diseases [5]. A number of evidences show that proteins in an oxidized state accumulate during aging processes [6]–[10]. In particular, it was inferred that oxidative protein damages are tightly linked to protein aggregation, which nowadays is considered one of the key processes determining pathologies like Alzheimer's, Huntington's and Parkinson's diseases [11]–[13]. Although the fundamental mechanisms at the basis of these pathologies are still unclear, it is now generally accepted that there is a strong causal link between protein aggregates, and in particular amyloid fibrils, and their aetiology [1], [2], [14]. Moreover, several reports suggest that protein modifications due to oxidation can be involved in these pathologies both as a secondary effect of a pre-existing dysfunction or as a main cause of the disease itself [8]-[12], [15]–[19].

Presence of reactive oxygen species (ROS) can induce irreversible structural damage and alter protein activities. Specifically, oxygen-containing radicals react with proteins leading to oxidized, degraded, and cross-linked molecules [15], [20] with cysteine, methionine, tryptophan, tyrosine and histidine being the most oxidation-prone residues [21], [22]. In particular, oxidation induces methionine conversion into methionine sulfoxide or sulfone derivatives [23]. Overall, oxidation of amino acids in protein polypeptide chains often reduces or modifies protein function, conformation and stability [24]–[26]. This is not surprising since even subtle modifications at single residue level in protein structures may alter specific inter-protein and protein-solvent interactions. In an energy landscape perspective [27], [28], even the oxidation of a single amino acid may change the energy funnel of the non-oxidized protein, both in terms of roughness and the ratio between different minima. Moreover, oxidation can also modify the energy potential surface leading to new and more stable minima. This brings to changes in both the aggregation propensity and/or pathways, including the formation of different aggregate morphologies with potentially different toxicity effects [10], [19], [29]–[34].

Uversky and co-workers have shown that fibrillation of α-synuclein at neutral pH is fully inhibited by methionine oxidation [29]. A similar behaviour was found for β-amyloid peptide [30], transthyretin [31] and apolipoprotein C-II [32], while an opposite tendency was observed for κ-Casein [34] and apolipoprotein A-I [33]. Notwithstanding the great interest in the interconnection between oxidation, post translational modifications, protein aggregation and age-related pathology, a deep understanding of the involved molecular mechanisms is out of reach.

HSA is the most abundant protein in mammalian plasma with multifunctional transport properties and it is widely used as a model system for studies on protein aggregation and amyloid formation [35]–[38]. It is well assessed that HSA can form amyloid fibrils in different solution conditions, and in particular at physiological pH [36].

Several evidences indicate that human serum albumin has significant antioxidant activity and may represent the main antioxidant in plasma [39], [40]. HSA is a single polypeptide protein of 585 amino acids whose heart-shaped three-dimensional organisation at neutral pH is composed by three homologous domains (I, II and III), each one consisting of two subdomains (A and B) with common structural elements. HSA secondary structure consists of about 67% α-helices and no β-sheets [41], [42]. At room temperature, tertiary structure is well defined: 17 disulphide bonds ensure some rigidity within each sub-domain but allow significant modification in the shape and size of the protein under different external conditions. At neutral pH the disulphide bridges are buried and not exposed to the solvent [42], [43]. HSA is also able to serve as a depot and transport protein in the circulation, because it can reversibly bind to a large number of endogenous and exogenous compounds. Two major drug-binding hydrophobic regions are located in domain II and domain III [44]. The most oxidation-sensitive aminoacids in this protein are the reduced Cysteine residue (Cys34) buried in a hydrophobic region in domain I and six Methionine residues (Met87 and Met123 in domain I; Met298 and Met329 in domain II; Met446 and Met548 in domain III) [40], [45]. It was proposed that methionines can act as endogenous antioxidants to protect cells from the accumulation of reactive oxygen species being the oxidation of these residues at the basis of antioxidant activity of proteins [22], [46], [47]. In particular, it has been suggested that oxidation of methionines and of Cys34 acts as scavengers of various reactive oxygen species [22], [48]. In principle methionine oxidation may affect the protein structure for sterical reasons. However, oxidation can also change the dominant forces, stabilising the native structure, e.g. by reducing side chain hydrophobicity, increasing of hydrogen bonding capacity, and by possibly modifying electrostatic and Van der Walls interactions. In this context, a prediction of the related changes in protein stability is difficult since the balance between the mentioned factors critically depends on the details of oxidised residues environment. As a consequence, in some cases protein conformation is slightly affected if solvent-accessible methionines on the protein surface are oxidized, while in other instances methionine modifications critically alter protein stability [46], [47].

Here we present an experimental study on the effects of hydrogen peroxide-induced oxidation on Human Serum Albumin (HSA) thermal stability and aggregation. Hydrogen peroxide (H_2_O_2_) is an oxidizing agent widely used as a model of ROS effects since it is easily generated in cellular environments in non-pathogenic conditions [49].

Our results indicate that, in our experimental condition, oxidation of HSA modifies the thermal stability of the protein structure against aggregation. Moreover, our data show that H_2_O_2_-induced oxidation reduces fibril formation propensity of HSA, suggesting that changes in the side chain due to oxidation vary the balance between critical intra- and inter-protein interactions and lead to different kinetics, intermediate species and final aggregates morphologies.

## Results and Discussion

### Spectroscopic Characterisation of Oxidised Human Serum Albumin

**Figure 1:**
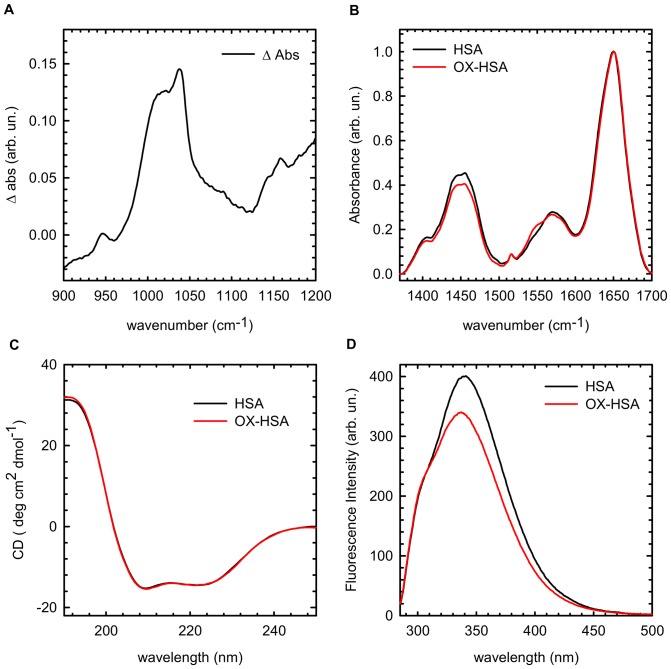
Spectroscopic Analysis of structural and conformational changes induced in human serum albumin by oxidation. **(A)** Difference FTIR absorption spectrum in the region 900–1200 cm^−1^. Samples were prepared in KBr pellet 5% w/w and signal of the HSA sample were subtracted to the OX-HSA in the same conditions; **(B)** FTIR spectra in the Amide region (1300–1700 cm^−1^) of HSA (black line) and OX-HSA (red line), 15 mg/ml in D_2_O. Data are normalized at Amide I' peak; **(C)** Far-UV CD spectrum of HSA (black line) and OX-HSA (red line) 0.5 mg/ml K-phosphate buffer at pH 7.4 at room temperature; **(D)** Intrinsic fluorescence spectra of HSA (black line) and OX-HSA (red line) 0.5 mg/ml K-phosphate buffer at pH 7.4 at room temperature. The excitation wavelength was 280 nm.

Spectroscopic properties of both HSA and oxidized HSA (OX-HSA) in solution at pH 7.4 were compared to obtain information about the effects induced on the protein structure and conformation by H_2_O_2_ oxidation. In Figure 1A the difference in the FTIR spectrum between OX-HSA and HSA sample in the same conditions is shown for the spectral region 900–1200 cm^−1^. In OX-HSA sample a significant peak is detected at 1044 cm^−1^ that it is a feature of methionine oxidation. It is well known that this residue is easily oxidized by hydrogen peroxide and converts into methionine sulfoxide [23] and the peak at 1044 cm^−1^ is assigned to S = O stretching vibration in methionine sulfoxide [50], [51]. Data in Figure 1A ensure that, in the present experimental conditions, treatment with H_2_O_2_ results in methionine oxidation. This result is in line with previous observations suggesting that H_2_O_2_ oxidation is specific for Cys and Met [52] and selectively modifies Cys34, Met123, Met298, Met446, and Met548 in HSA structure [53].

Information on the effect of oxidation on the secondary and tertiary structure can be retrieved by comparing the FTIR spectrum of HSA and OX-HSA in the amide region (Figure 1B), far UV CD spectra (Figure 1C) and protein intrinsic fluorescence (Figure 1D).

In panel 1B the normalized FTIR spectra of HSA (black line) and OX-HSA (red line) are reported. In both cases protein is dissolved in D_2_O to a final concentration of 15 mg/ml. No appreciable differences between the two samples in the amide I' region (1600–1700 cm^−1^) can be detected. The amide I' absorption gives information on protein secondary structures. Figure 1B indicates that HSA and OX-HSA secondary structures are nearly identical and the peak at 1654 cm^−1^ confirms that their secondary structure is mainly formed by α-helices [54]. On the contrary, a clear difference is evident in the region of the Amide II (1550 cm^−1^) and Amide II' (1450 cm^−1^). The amide II/II' band is particularly sensitive to the H/D exchange of amide groups and may therefore probe protein structural compactness and flexibility [54], [55]. In particular, H/D exchange in the amide II region gives qualitative information on the amount of hydrogen atoms within the core of the protein and inaccessible to the solvent. The ratio between the two peaks is different for HSA and OX-HSA and, in particular, H/D exchange is more pronounced for native HSA as the Amide II' (1450 cm^−1^) intensity shows. This result suggests that protein conformation is different in the two samples and it is possible to infer that oxidation process induces a reduction of the solvent accessibility to the internal parts of protein molecules, possibly leading to a more compact structure.

In Panel 1C the far-UV CD spectra of HSA (black line) and OX-HSA (red line) are reported. In both cases, protein is dissolved to a final concentration of 0.5 mg/ml in K-phosphate buffer at pH 7.4. These measurements are in agreement with results on secondary structure reported in panel 1B. Specifically, the two spectra are superimposed and their shapes are characterised by two minima, one at 208 and other at 222 nm, confirming that the helical structure [35], [36], [38], within experimental error, remains unmodified after oxidation. Near-UV CD measurements on the same samples were also performed (see Figure S1 in File S1). Also in this case, no significant differences are observed between HSA and OX-HSA at RT, suggesting that the oxidation does not change the chiral environment of aromatic residues. This is not in contrast with H/D exchange measurement in the Amide II region which gives information on the amount of hydrogens still present within the core of the protein and allows highlighting the increased compactness of OX-HSA. Subtle changes in the protein tertiary structure can be singled out by fluorescence measurements.

In Figure 1D intrinsic fluorescence emission spectra (λ_exc_ = 280 nm) are reported for HSA (black line) and OX-HSA (red line). Samples are in the same conditions as for the far-UV CD measurements. As can be seen, a significant difference between the two spectra exists. In these conditions, HSA intrinsic fluorescence is mainly attributable to the unique Tryptophan residue at positions 214 (Trp214) located in a hydrophobic pocket of domain II [41], together with a small contribution of tyrosines (see Figure S2 and S3 in File S1 for details) [56]. As a consequence, observed changes in the fluorescence signal mainly reflect structural modification at the level of protein tertiary structure due to oxidation effects. In particular, OX-HSA fluorescence intensity is reduced and slightly blue-shifted. The position of Tryptophans' emission band indicates the extent of solvent exposure of the chromophores and a blue-shift may stem from a less polar environment. As a consequence, data in Figure 1D indicate a reorganization of protein chain into a more tightly packed structure, with a decreased accessibility to the solvent [57]. This suggests a more compact environment of Trp214 within domain II and/or an increased hydrophobicity of the area in its surroundings. It is worth nothing that, by oxidation, Tryptophans can in principle undergo chemical modifications and, for instance, form one of their metabolites e.g. kynurenine (Kyn214). This would lead to significant variations in the absorption and fluorescence spectrum [58]. However, no significant changes were measured in the absorption spectrum of the two samples, which are perfectly superimposable (data not reported). This allows us to rule out significant alterations in chemical structure of Trp214. Importantly, the lower flexibility of the internal part of OX-HSA suggested by the data in Figure 1D is in line with our FTIR results.

**Figure 2:**
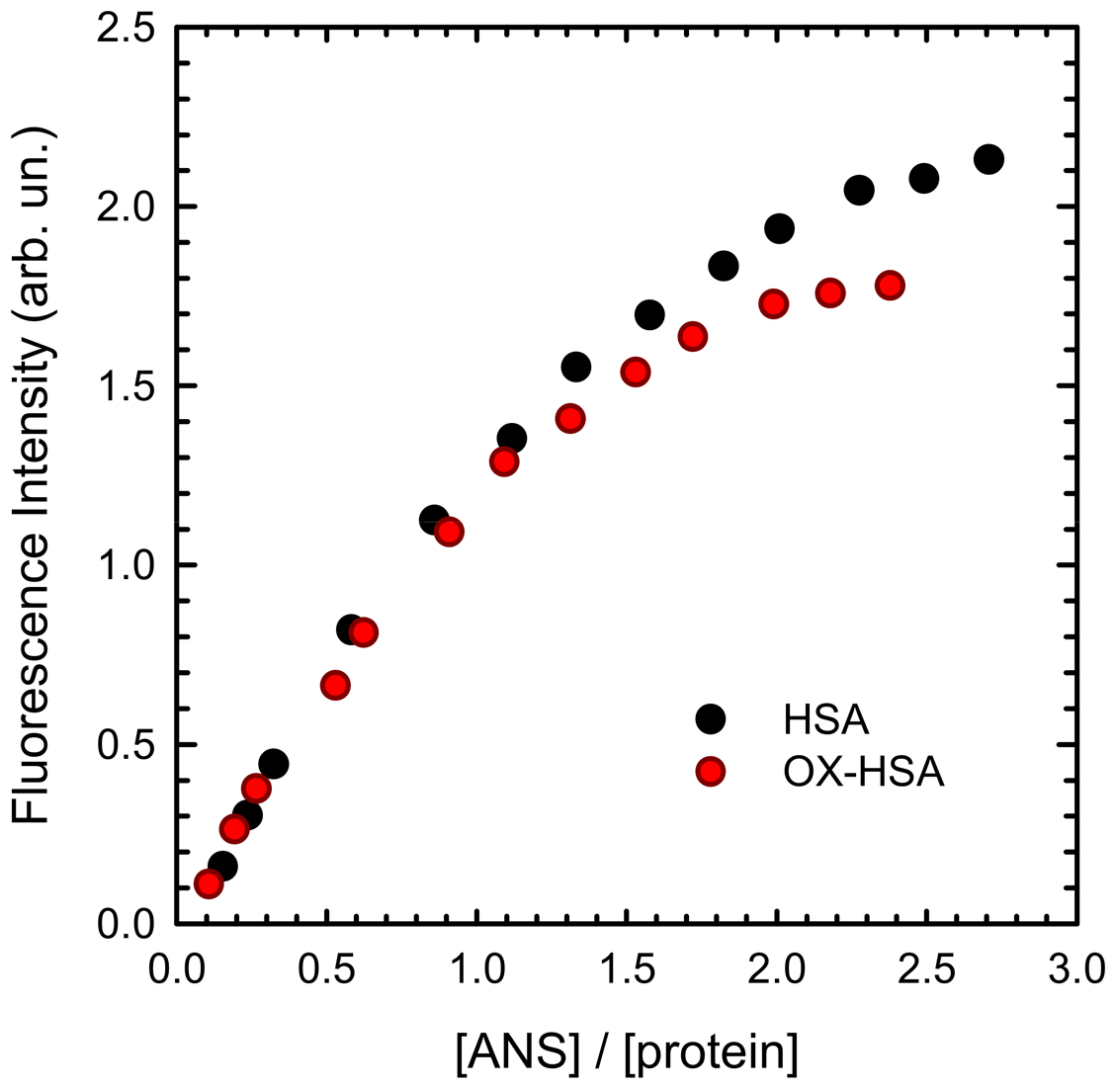
ANS titration curves. Integrated intensity of ANS emission as a function of molar ratio [ANS]/[protein] for HSA (black) and OX-HSA (red) in the range [ANS]/[protein]  = 0.1–2.8. ANS fluorescence was measured at room temperature using an excitation wavelength λ_exc_ = 380 nm in HSA and OX-HSA samples 0.5 mg/ml in K-phosphate buffer 0.1 M at pH 7.4

In order to gain further insights on conformational changes due to oxidation, we analysed fluorescence spectra variations of Anilino-Naphthalene-Sulfonic acid (ANS) as extrinsic dye. In particular, the binding of HSA and OX-HSA to ANS was analysed by means of a titration experiments whose results are reported in Figure 2. ANS fluorescence is used to probe protein conformational reorganizations associated with changes in the solvent exposure of hydrophobic regions [59], [60], as well as to detect the formation of molten globule-like species [61] and characterize non-polar surface patches of proteins. An increase in the fluorescence intensity and a blue-shift on the emission spectrum are usually observed upon binding of ANS to either exposed hydrophobic region or hydrophobic clusters [35], [59], [62]–[64].

Figure 2 shows the integrated emission spectrum of ANS as a function of increasing [ANS]/[protein] molar ratio for both HSA (black dots) and OX-HSA (red dots) samples. No significant variations in the emission band shape were observed. ANS intensity shows a similar trend for both samples, with a first monotonic increase and then a plateau. On the contrary, a difference between the two samples can be appreciated in the intensity growth as a function of the [ANS]/[protein] ratio. It is known that, in native HSA structure, two ANS binding sites are present: one with larger affinity located in domain III and a lower-affinity site in domain II [44], [56], [60], [65]. The presence of these two sites determines a well-known biphasic behaviour in the titration curves. Specifically, up to a molar ratio [ANS]/[Protein] of about 0.65, ANS mainly interacts with the higher affinity binding site located in the domain III, while, at higher ANS concentration, the dye occupies both binding sites [44]. In our experiments up to [ANS]/[Protein] ∼0.65, no relevant differences are observed between the two samples. Significant differences are instead observed for higher molar ratios, in which the binding site situated in domain II is also involved. These data suggest that oxidation induced by hydrogen peroxide mainly affects HSA within regions located in domain II with respect to the ones in domain III. This could be explained in terms of reduction in accessibility of hydrophobic sites of HSA in this domain due to oxidation. In particular since Met298 is one of the most oxidation-prone sites within the protein structure [53], it is likely that H_2_O_2_ oxidation is effective on this methionine. Such a residue is located in proximity of ANS binding site in domain II. As a consequence, the addition of an oxygen atom to the sulphur of these methionine residues may decrease the hydrophobicity of the side chain, leading to a reduction of the ANS affinity in this area as revealed by the data in Figure 2. This scenario would also match with previous studies in which both H_2_O_2_ and Cloramine T were used as oxidation agents [45], [48]. Moreover, structural studies show that HSA, after mild oxidation treatment, has higher resistance against heating stress, and it is less flexible than the native non-oxidized protein. Such changes also cause alterations of albumin binding properties to physiological ligands [66]. Modifications due to methionine oxidation may also lead to a structural reorganisation toward a more compact structure or the presence of oxygen molecules may sterically hider hydrophobic pocket accessibility. All these evidences are in line with results obtained by intrinsic fluorescence and FTIR data in the Amide II region.

Overall, data in Figure 1 and 2 clearly show that oxidation of HSA does not significantly modify protein secondary structure but induces significant changes in protein conformation. In particular, oxidation leads the internal part of the protein to be less accessible to the solvent as monitored by H/D exchanges and intrinsic fluorescence, without dramatically changing chiral properties of aromatic residues environment. Our data confirm the formation of methionine sulfoxide as an oxidation product, in line with independent reports suggesting methionine and Cys34 as the most susceptible residues for hydrogen peroxide oxidation [53]. Our data do not give specific information on modifications potentially occurring in domain I and due to possible Cys34 oxidation. However, ANS data clearly suggest that significant modifications occur in the proximity of domain II leading to an enhanced compaction of the protein and a reduced solvent accessibility for the ANS binding site in this region.

### Effect of oxidation on HSA thermally induced supra-molecular aggregation

**Figure 3:**
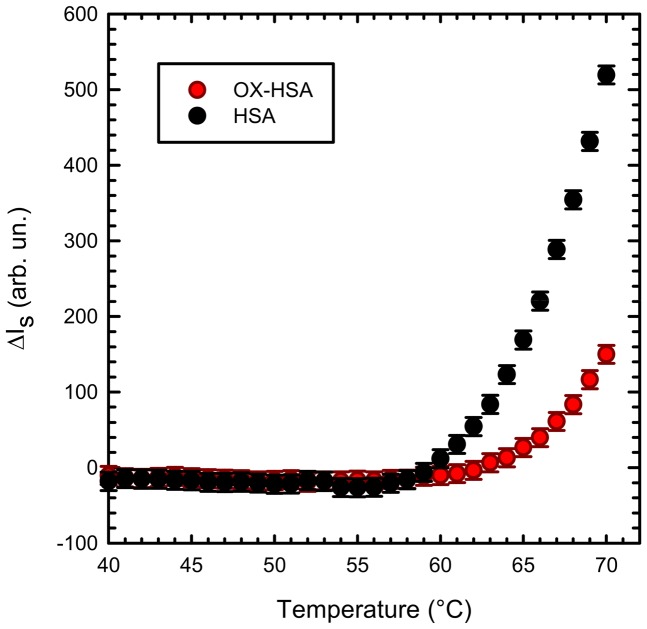
Aggregation propensity for HSA and OX-HSA. Rayleigh scattering variations as a function of temperature measured during an upward temperature scan at 12°C/h for HSA (black) and OX-HSA (red) samples 0.5 mg/ml in K-phosphate buffer 0.1 M at pH 7.4.

In Figure 3 we report the variations in Rayleigh scattering intensity during an upward temperature scan (scan rate 12°C/h) of HSA (black) and OX-HSA (red) samples at a protein concentration of 0.5 mg/ml. For both samples, an increase in the scattering intensity is observed above a certain temperature, indicating the onset of an aggregation process. However, the critical temperature for the activation of the aggregation process appears to be higher for OX–HSA sample and it is also possible to note that, above such temperatures, the slope of the scattering growth is steeper for HSA sample. The observed differences can be taken into account as the result of different protein thermal stability and aggregation tendency. In particular the OX-HSA sample undergoes aggregation process at higher temperature with respect to the native molecule in the same conditions.

In the light of the results presented in the previous section, data in Figure 3 are not surprising. The enhanced protein stability/structure compactness induced by the oxidation treatment of HSA may alter the balance between fundamental interactions, e.g. long range electrostatic forces, Van der Waals, hydrogen bonding and hydrophobic interactions, also resulting in a reduced aggregation propensity [67], [68]. Moreover, structural constraints imposed by oxidation can also have a role. In fact, they can slightly alter the monomer conformation and change protein-solvent interactions, influencing conformational changes during the aggregation process. Intra- and inter-molecular hydrophobic interactions are probably modified by the more hydrophilic methionine sulfoxide side chain, affecting the aggregation process [32].

It is well known that in suitable conditions HSA is prone to aggregation and that the aggregation process may lead to different aggregate species, e.g. oligomers, amorphous aggregates, small curly fibrils or mature amyloid fibrils [36]–[38], [69]. In particular, at physiological pH and high temperature, HSA forms amyloid fibrils via a downhill process. The reaction does not imply nucleation mechanisms [36], [38] suggesting that the conformational change is the rate limiting step for the formation of amyloid fibrils.

**Figure 4:**
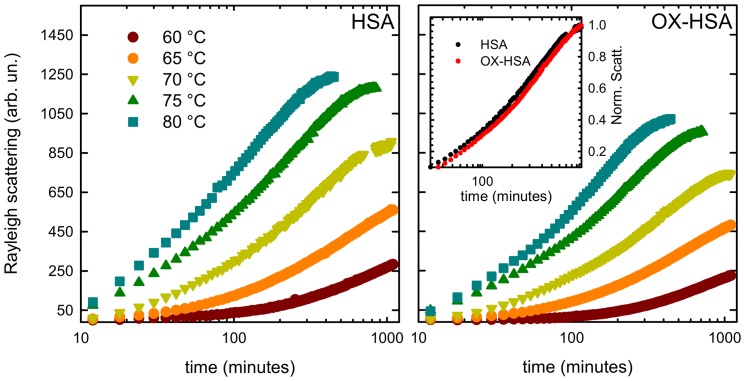
Temperature effect on aggregation kinetics. Temporal evolution of Rayleigh scattering intensity measured at λ = 280 nm for HSA **(A)** and OX-HSA **(B)** 0.5 mg/ml in K-phosphate buffer 0.1 M pH 7.4 during isothermal incubation at 60, 65, 70, 75 and 80°C. The inset shows the superimposed kinetics at 70°C when scaled for the Rayleigh scattering plateau value.

Data in Figure 3 suggest that the aggregation process is somehow hindered by oxidation. To further investigate the effect of oxidation on HSA supra-molecular assembly at different length and time scales, we performed isothermal kinetic experiments. In Figure 4, temporal evolution of Rayleigh scattering intensity during incubation of HSA (Figure 4A) and OX-HSA (Figure 4B) 0.5 mg/ml in phosphate buffer pH 7.4 at different temperatures (60, 65, 70, 75 and 80°C) is presented. The intensity both for HSA and OX-HSA grows, suggesting that the size of scattering objects in solution increases as a function of time. Moreover, in agreement with results in Figure 3, data in Figure 4A and 4B qualitatively show that the scattering intensity at the end of the kinetics measured in OX-HSA samples is lower compared to the correspondent HSA ones. This heuristically suggests that, in average, the size of OX-HSA aggregates is smaller than the HSA ones. For both samples, aggregation process appears to be highly temperature dependent, being faster at higher temperature. Moreover, is worth nothing that, notwithstanding aggregation kinetics profile are very similar, it is not possible to superimpose them using suitable scaling factors both in the intensity and in the time scale as otherwise reported for others fibrillating proteins [70]–[73]. This suggests that aggregation mechanisms underlying supra-molecular assembly are different. Moreover, the OX-HSA aggregation process is slightly slower compared to the HSA one. This can be appreciated in the inset shown in Figure 4B, where aggregation kinetics at 70°C is reported after normalisation to plateau value.

### Effect of oxidation on conformational changes

**Figure 5:**
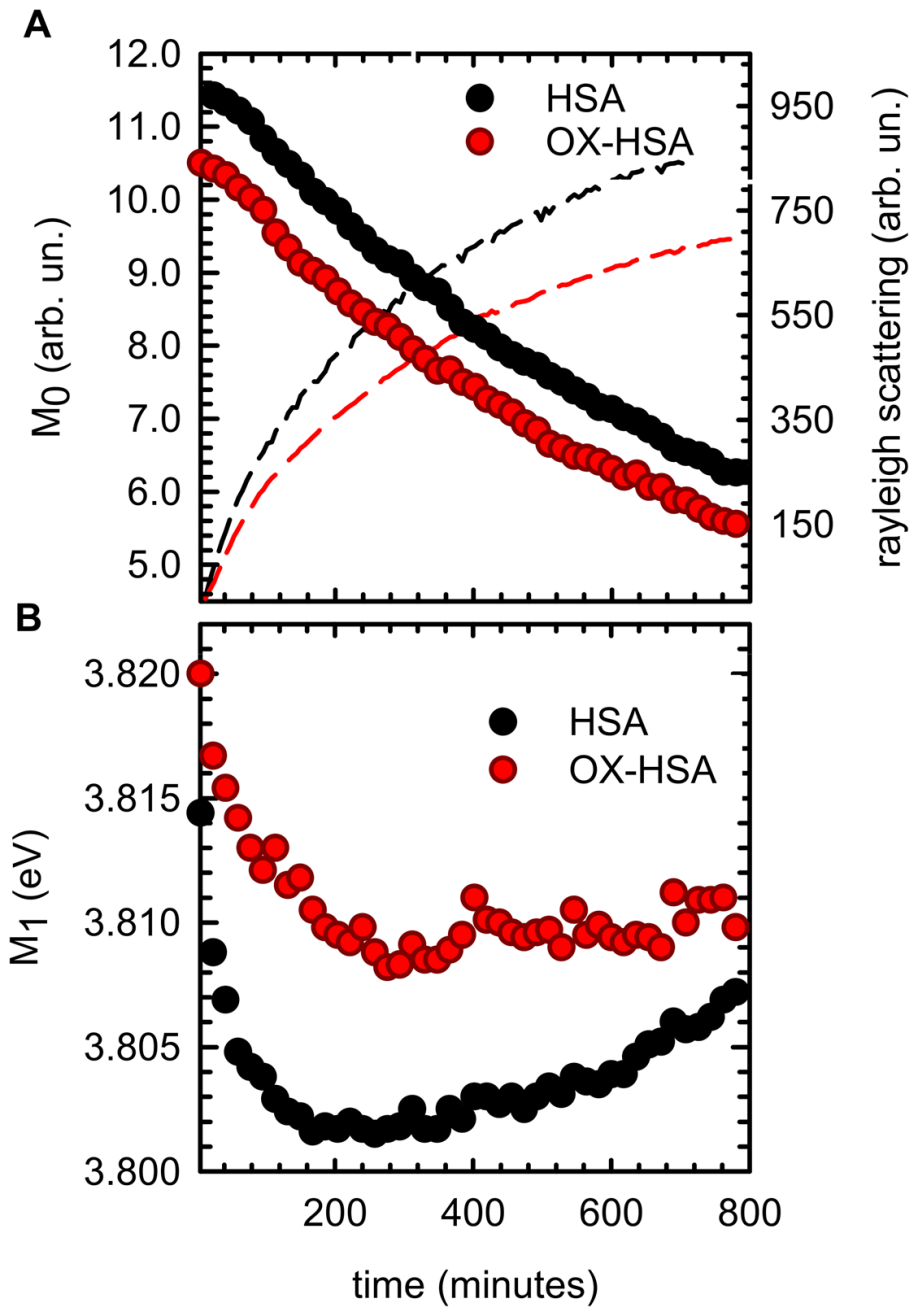
Tertiary structure changes during the aggregation process. Temporal evolution of intrinsic fluorescence emission band spectral moments for HSA (black dots) and OX-HSA (red dots) samples 0.5 mg/ml in K-phosphate buffer 0.1 M at pH 7.4 during isothermal incubation at 70°C. **(A)** Zeroth moment (M_0_) (dots) and Rayleigh scattering (solid line) as a function of time. **(B)** First moment as a function of time. Rayleigh scattering and fluorescence spectra were acquired under excitation at λ_exc_ = 280 nm.

In all the observed aggregation kinetics no lag-phase is observed, suggesting the absence of any nucleation mechanisms. This is in line with results on HSA aggregation in similar conditions that suggest a downhill process [36]–[38]. However, potential differences in the observed aggregation mechanisms induced by protein oxidation could be singled out following the conformational changes that both HSA and OX-HSA undergo during the process. In Figure 5 we report the analysis of HSA and OX-HSA intrinsic fluorescence changes during the aggregation process at 70°C. Fluorescence emission spectra obtained under excitation at 280 nm were analysed in terms of spectral moments [74] and the temporal evolution of the zeroth moment (M_0_) and of the first moment (M_1_) for HSA (black dots) and OX-HSA (red dots) is reported in panel 5A and 5B, respectively. The observed fluorescence intensity can be mainly ascribed to Trp214 signal [44], [75], with a minor contribution from tyrosine. Due to tryptophans' high sensitivity to local environment, changes in their emission account for subtle modifications in protein structures occurring in the proximity of these residues [56].

The value of M_0_ is representative of the fluorescence emission intensity and it shows for both samples a monotonic decrease during the time of observation. In panel 5B the temporal evolution of M_1_ is also reported, being a measure of the position of the band [74]. It is well known that tryptophan emission quantum yield may either decrease or increase upon conformational changes in the surroundings of these chromophores and that red-shift of the bands usually indicates a more polar environment [56], [76]. These changes are generally related to protein aggregation and amyloid formation in several studies [77]–[82]. In the early phases of the aggregation process, M_0_ for OX-HSA sample is lower compared to the one for HSA. This reflects the same difference observed at room temperature (Figure 1D), indicating a minor exposure to the solvent. Moreover, this matches with the peak position for OX-HSA being slightly blue shifted compared to HSA (panel 5B). During the aggregation process the M_0_ value decreases as a function of time with similar features for both OX-HSA and HSA samples. Conversely, differences between the two samples are detected in the temporal evolution of M_1_. A first red shift is observed for both samples and then, after about 200 minutes, a blue shift occurs, being more pronounced for the HSA sample. These data suggest the following scenario: during supra-molecular assembly, an initial (200 minutes) exposure to the solvent of Trp214 in domain II occurs in both samples, and then Trp214 environment undergoes a reorganization into more tightly packed structures with a decreased accessibility to the solvent. This latter effect is more pronounced in the HSA aggregation process, suggesting that the oxidation leads to different intermolecular interactions during the aggregation process compared to the untreated HSA sample.

**Figure 6:**
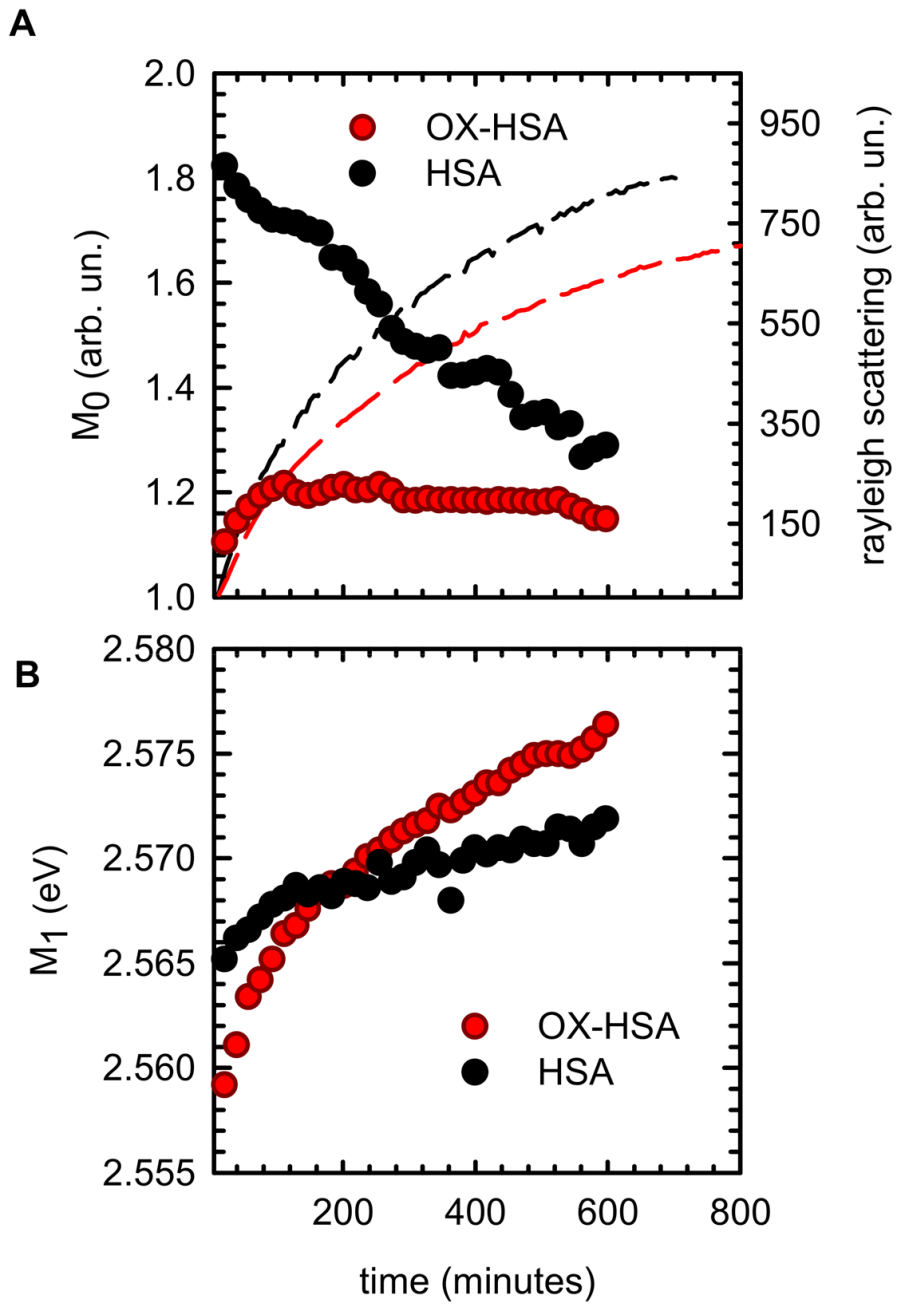
Changes in hydrophobic regions during the aggregation process. Temporal evolution of ANS fluorescence emission band spectral moments for HSA (black dots) and OX-HSA (red dots) samples 0.5 mg/ml in K-phosphate buffer at pH 7.4 during isothermal incubation at 70°C. **(A)** Zeroth moment (M_0_) (dots) and Rayleigh scattering (solid line) as a function of time **(B)** First moment as a function of time. Spectra were acquired using [ANS]/[protein] concentration ratio 0.3 in order to single out information on domain III changes. ANS Fluorescence spectra were acquired under excitation at λ_exc_ = 380 nm. Rayleigh scattering was acquired under excitation at λ_exc_ = 280 nm.

To better clarify the role of hydrophobic interactions involved in the aggregation processes, and the potential oxidation-induced effects on them, we followed the temporal evolution of ANS fluorescence emission. In Figure 6 we report the temporal evolution of M_0_ (panel A) and M_1_ (panel B) relative to ANS fluorescence emission at 70°C in HSA (black dots) and OX-HSA (red dots) sample. The fluorescence emission kinetics of ANS dye was measured at 0.3 [ANS]/[protein] molar ratio. At this low ANS concentration, the aggregation process is not affected by the presence of the dye.

ANS can be used to monitor structural changes involving hydrophobic regions during aggregation processes [35], [61], [73], [74], [83], [84]. These regions are either already present in native protein structures or formed during the evolution of the aggregation. The enhancement of ANS fluorescence intensity indicates an increased interaction of the dye with suitable hydrophobic binding sites and this increase is usually accompanied by a blue shift of the fluorescence emission band. As reported in the previous section, it is well assessed that there are two main ANS binding sites in HSA: the first with larger affinity located in domain III and the second with a lower affinity located in domain II, where Trp214 is also located. The molar ratio [ANS]/[protein] = 0.3 used here enables us to single out information on the conformational changes mainly involving domain III [44], [65], [85]. While M_0_ in HSA sample shows a progressive decrease, the M_0_ in OX-HSA initially increases reaching a plateau in about 200 minutes. In both HSA and OX-HSA, the value of M_1_ monotonically increases (blue shift) indicating progressive changes within the internal hydrophobic zones and, specifically, in the surroundings of domain III. Interestingly, notwithstanding the ANS intensity measured at RT for the two samples is the same (see Figure 2), the intensity value measured at 70°C at the beginning of aggregation process is significantly lower for OX-HSA sample compared to HSA one. This suggests that oxidation induces conformational changes in the proximity of ANS binding sites in domain III that are of different nature/extent compared to the ones in the untreated sample. One can argue that OX-HSA and HSA molecules undergo different partial opening of the structure in proximity of ANS binding sites, exposing different reactive areas to the solvent. After that, in the first phase of aggregation, new binding sites for this hydrophobic dye are progressively formed/accessible. The ANS fluorescence band behavior is more complex in HSA sample. Specifically, a larger affinity for this dye is maintained even at high temperature, being this probably due to the persistence of the main structural characteristics of the binding site in domain III. Then, ANS intensity decreases with a biphasic trend during the whole aggregation process. A first decrease is observed in a similar temporal range as the ANS growth in OX-HSA sample, and a further linear decrease continues in the second step of aggregation, while a plateau is observed in OX-HSA. Unexpectedly, ANS peak undergoes a small blue shift for both samples. This would indicate the interaction of the dye with more affine environment and may be the result of multiple protein-dye interactions within different environments experienced by dye molecules. It is possible to infer that the overall change in the fluorescence signal is due to two simultaneous effects: the partial opening of ANS domain III (i.e. decrease of the signal) and the interaction of ANS molecules with new binding sites (i.e. increase of the signal). This would explain the observed blue shift due to the generation of new hydrophobic areas during the aggregation process. Overall, these results suggest that domain III surroundings are strongly affected by the oxidation treatment, heavily shifting the balance between attractive and repulsive interactions among partially unfolded protein molecules. This leads to the differences between the aggregation processes of HSA and OX-HSA.

**Figure 7:**
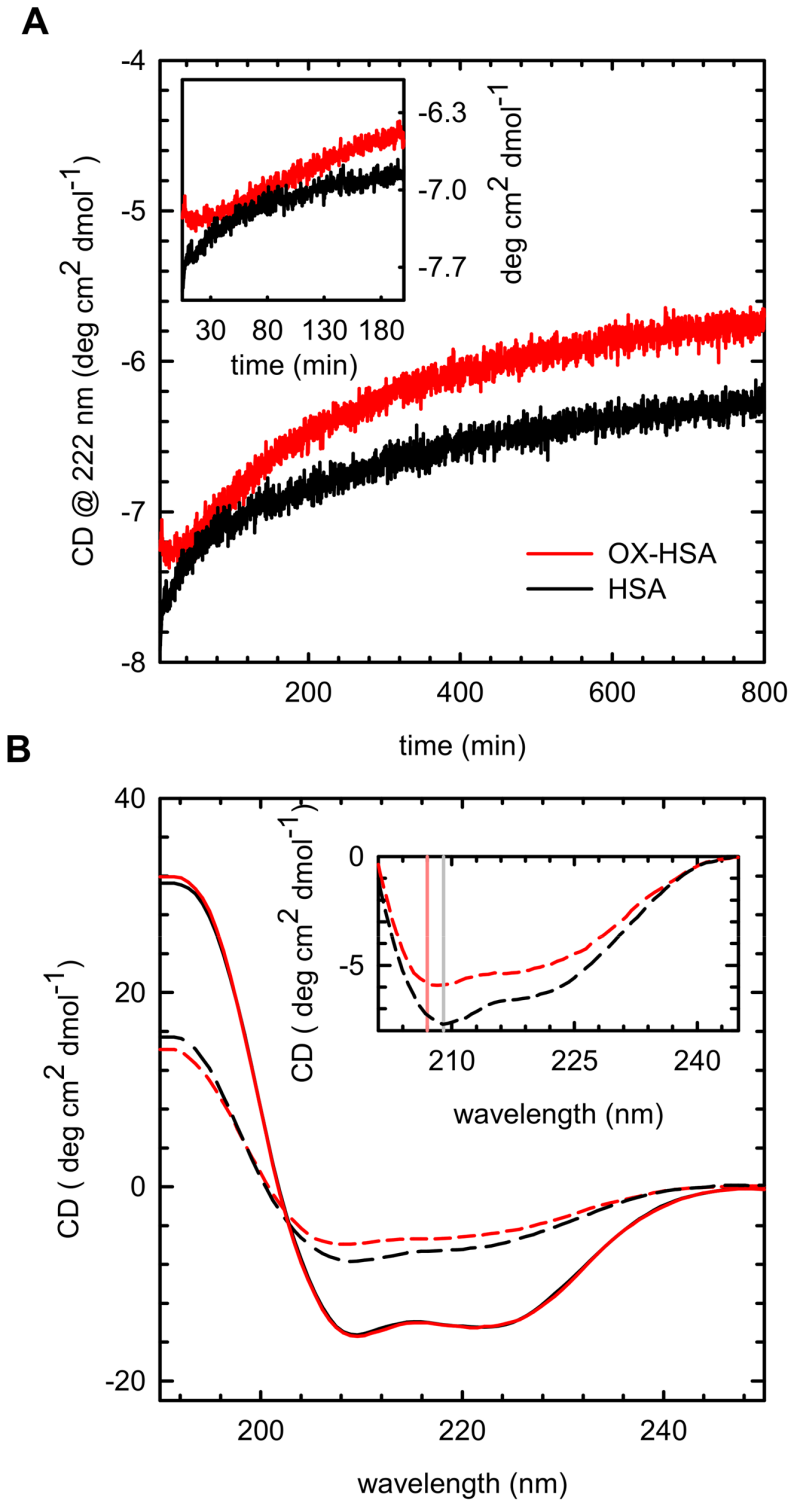
Secondary structure changes. **(A)** Temporal evolution of CD signal measured at 222 for HSA (Black line) and OX-HSA (red line) sample 0.5 mg/ml in phosphate buffer 0.1 M at pH 7.4 incubated at 70°C for 800 minutes. Inset: magnification of the early stages **(B)** Far-UV CD spectra at room temperature of freshly prepared HSA (solid black line) and OX-HSA (solid red line) sample and of the same samples of HSA (dashed black line) and OX-HSA (dashed red line) after 800 minutes of incubation at 70°C. Inset: magnification of significant differences in the spectra. Solid lines at 207 (red) and 209 (grey) nm are guides for eyes.

In order to analyse secondary structure changes of the observed aggregation process, we followed Far UV-CD spectrum changes as a function of time. Figure 7A reports the temporal evolution of CD signal at 222 nm for HSA (black line) and OX-HSA (red line) during incubation at 70°C. We also report in Figure 7B the far-UV CD spectrum measured at RT for HSA (dashed black line) and OX-HSA (dashed red line) samples after 800 minutes of incubation at 70°C compared with the spectra of freshly prepared samples (solid lines).

As can be seen, at 70°C the CD signal at 222 nm progressively increases as a function of time in both samples, indicating that changes in secondary structure occur during the aggregation process. It is worth noting that, although they have similar extents, the observed changes follow different temporal evolution, especially in the first 200 minutes (see inset in Figure 7A). As shown in Figure 7B, the CD spectra of freshly prepared samples are almost super imposable and they present the typical shape with two minima at 208 nm and 222 nm, indicating a high α-helical content. The aggregation process leads to a reduction of ellipticity in both samples, while differently affecting their shapes (see inset in Figure 7B). This indicates a partial conversion of α-helices into β-structures and loops with different extents. Similar changes were observed in Serum Albumin aggregation processes and they were attributed to the formation of intermolecular β-structures during fibrillar assembly [36], [71]. In line with previous results, these aggregates appear to maintain part of their native α-helical structure [36], [71], [86]. Importantly, structural changes observed in this low concentration regime are in agreement with analogous results obtained at higher protein concentration and measured by FTIR spectroscopy (see Figure S4 in File S1).These results could suggest that aggregate formation proceeds with different pathways. These are characterized by different secondary and tertiary structure leading to the formation of species with different molecular structures. This is confirmed by Thioflavin T (ThT) [87]–[89] assay that shows a significantly higher fluorescence emission for the HSA compared to the OX-HSA, suggesting a more amyloid-like nature for the HSA aggregates (see Figure S5 in File S1).

Overall, the data reported in this section clearly indicates that oxidation of HSA induces changes in protein conformation bringing to an increased protein resistance against thermal aggregation. In the same experimental conditions, the two proteins undergo aggregation processes via different aggregation pathways, involving different conformational changes of the whole protein. We were able to elicit that domain III in HSA and OX-HSA is modified by oxidation and has different roles in the observed aggregation. In particular, the hydrophobic interactions contributing to molecular assembly appear critically different. Moreover, CD measurements and ThT assay evidence that the aggregated species have a different nature.

**Figure 8:**
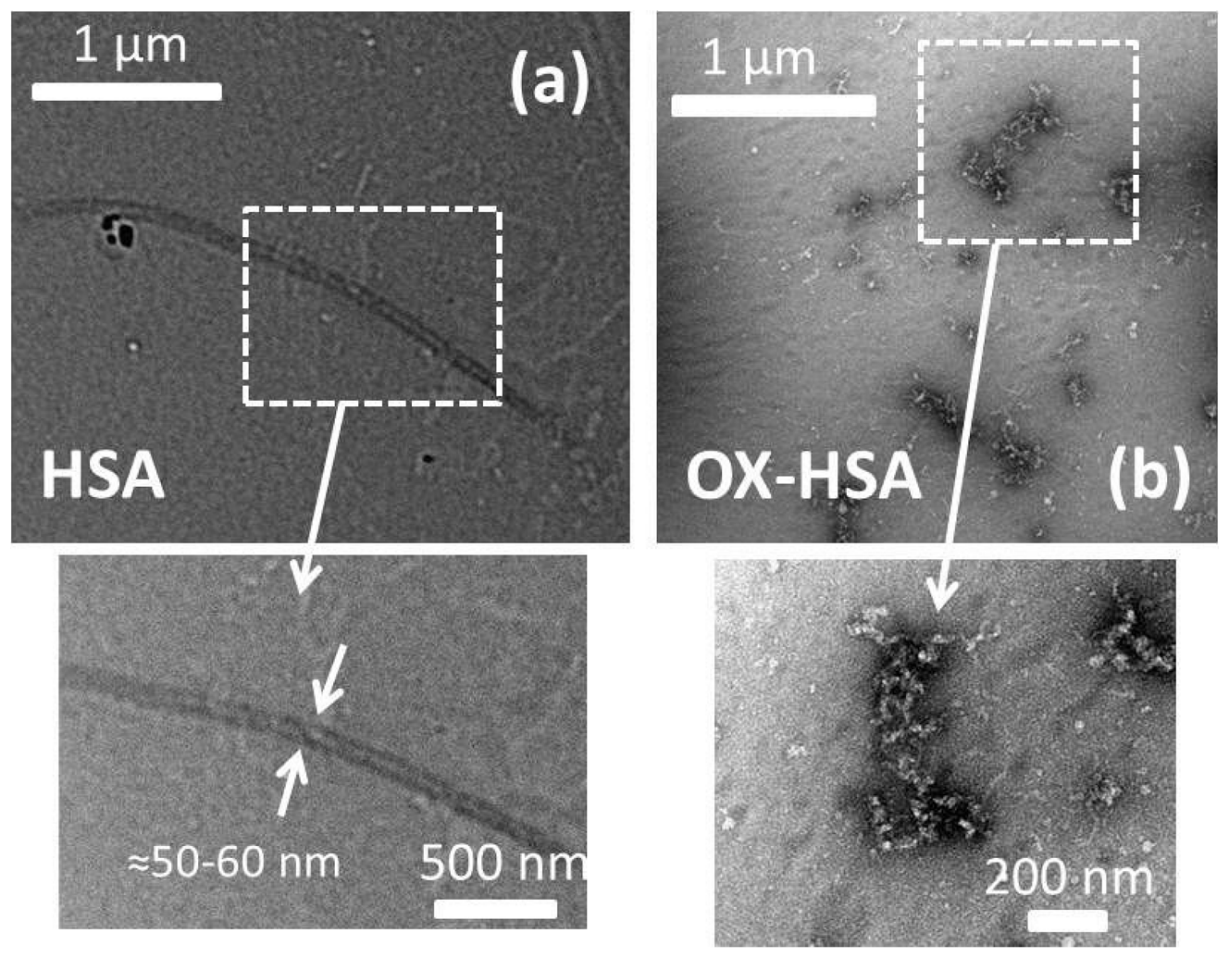
Morphologies of HSA and OX-HSA aggregates. TEM images of HSA (a) and OX-HSA (b) aggregates obtained upon thermal incubation 10 mg/ml protein solution in K-phosphate buffer 0.1 M at pH 7.4 at 70°C for 12 hours.

To further corroborate the effect of oxidation in modifying the aggregation pathway of HSA, we imaged final aggregates obtained incubating 10 mg/ml HSA and OX-HSA in K-phosphate 0.1 M at pH 7.4 at 70°C for 12 h by means of transmission electron microscopy (TEM). TEM images of HSA and OX-HSA aggregates are shown in Figure 8A and 8B respectively, with the only aim to qualitative evaluate overall differences in the morphology of the aggregates. Importantly, an increased protein concentration certainly would lead to a different aggregation process, yet evidencing the dramatic effect of H_2_O_2_-induced HSA oxidation on the aggregates nature and morphology.

As can be seen HSA aggregation process leads to a clear formation of large fibrillar structures with a linear rod-like morphology. Such structures are distributed within the whole sample. Conversely OX-HSA aggregates are composed of compact clusters of amorphous aggregates and worm-like aggregates while the presence of elongated objects is, in this case, not detected.

## Conclusions

In this work we have shown that hydrogen peroxide-induced oxidation modifies HSA conformation increasing its resistance against thermal aggregation. Subtle modifications in the initial state, due to mild oxidation, drive the aggregation routes of oxidized molecules toward different final aggregate morphologies with a reduced fibrillar nature.

We identified the oxidation of amino acids as the main responsible of changes in the protein tertiary structure that alter the balance between different intermolecular interactions. Spectroscopic measurements reveal an increased compactness of the oxidized protein with reduced accessibility to the solvent of HSA domain II environment. Such changes, which do not affect HSA secondary structure, can be mainly attributed to the oxidation of specific methionine residues. The oxidation of these residues was assessed by FTIR spectroscopy in the 900–1200 cm^−1^, which clearly shows the formation of methionine sulfoxide with a larger and more polar side chain and with an increased capacity of creating hydrogen bonding. Methionine oxidation prevents amyloid fibril formation via a partitioning of aggregation pathway involving domain III. In fact, while similar conformational changes for both proteins occur in domain II during the first part of the aggregation process, a sudden partial unfolding in domain III occurs only in oxidized protein, with consequent exposure of hydrophobic regions. These regions are involved in the supra-molecular assembly via the formation of new hydrophobic clusters. This fast formation prevents, in the second part of the process, an ordered reorganization of Trp214 surroundings into more tightly packed structures (Figure 6B). The different conformational changes in domain III possibly modulate also modifications in secondary structures which result in different temporal evolution and final content of intermolecular β-structures.

The results presented in this work have a twofold relevance. On one hand, they highlight, from a general point of view, the importance of methionine residues as modulators of fibril formation [29]–[34] adding evidences of the implication of this post translational modification in the aggregation process. On the other hand, it gives information on the antioxidant properties of Human Serum Albumin: under mild oxidation conditions this protein interacts with hydrogen peroxide acting as a scavenger [39], [40], [46], [47] and, as a result, its structure becomes more compact and stable against aggregation. It should be also noted that oxidation affects the ANS binding site in domain III with consequent possible implications in its function as transport protein. Finally, oxidation also modifies the aggregation pathway of HSA leading to aggregate species with different molecular properties and morphologies with possible different cytotoxic effect.

## Materials and Methods

### Sample preparation

Human Serum Albumin (HSA), Anilino-Naphthalene-Sulfonic acid (ANS), Thioflavin T (ThT) and Hydrogen Peroxide 30% solution w/w (H_2_O_2_) were purchased from Sigma. Oxidation of HSA was induced by incubating (2 mg/ml) HSA in a 3% w/w H_2_O_2_ solution for 24 h at 25°C. Non-reacted peroxide was removed from the protein solution by means of extensive water dialysis. To minimize additional oxidation during the dialysis, the dialysis was performed in cold room at 7°C. After dialysis the sample was lyophilized and stored at −20°C. During the incubation, to monitor the oxidation process, absorption in the range 240–400 nm and intrinsic fluorescence mainly related to Trp214 were measured and no significant variations were observed in the absorption spectra whereas the intrinsic fluorescence displayed a progressive decrease until a plateau value. OX-HSA state was probed by mass spectroscopy measurements (see Figure S6 and S7 in File S1).

### Fluorescence measurements and Rayleigh scattering

All the measurements on HSA and oxidized HSA (OX-HSA) were performed in K-phosphate buffer 0.1 M at pH 7.4. Protein solutions were freshly prepared and filtered through a 0.20 µm filter just before the measurements. Protein concentration was determined by UV absorbance and concentration was approximately 7 µM. Concentration measurements were performed by means of a Shimadzu-240IPC UV-Vis spectrophotometer. Protein concentration was obtained using absorption at λ = 280 nm, and a molar extinction coefficient ε = 36500 cm^−1^ M^−1^.

Intrinsic fluorescence emission spectra were detected using a Jasco FP-6500 equipped with a Jasco peltier thermostat; samples were positioned in a cuvette of 1 cm and all emission spectra were recorded at 0.5 nm wavelength intervals with excitation and emission band-width of 3 nm, scan-speed of 100 nm/min and integration time of 1s. ANS and ThT fluorescence emission spectra detected using a Jasco FP-8500 equipped with a Jasco peltier thermostat; samples were positioned in a cuvette of 1 cm and all emission spectra were recorded at 0.5 nm wavelength intervals with excitation and emission band-width of 5 nm and 2.5 nm respectively, scan-speed of 100 nm/min and integration time of 1s. To avoid artifacts, during aggregation kinetics control absorption and fluorescence excitation measurements were performed before and after each thermal treatment.

During kinetic experiments, after thermal equilibration, the intrinsic emission spectra were obtained under excitation at 280 nm in the range 270–550 nm every 6 minutes. Excitation spectra at 25°C, before and after the kinetics, were measured to control possible variations of the excitation band profile. Simultaneously, with emission spectra, Rayleigh scattering intensity at 90° was also measured as the maximum of the elastic peaks of excitation light. The choice of reporting kinetics measurements obtained at excitation wavelength λ = 280 nm, where a small tyrosine contribute is present, constitutes a good compromise between the need of maximizing tryptophan signal and to minimize scattering contribution in fluorescence signal. This allows a reliable analysis of band shape and intensity. RT excitation and emission spectra of HSA and OX-HSA are reported in File S1.

After each aggregation kinetics ThT was dissolved in the sample at 40 µM concentration. ThT emission spectra were detected under an excitation wavelength of λ_exc_ = 440 nm in the range 445–650 nm.

Room temperature titrations with ANS were made manually by adding small aliquots of concentrated ANS solution to protein solution. HSA concentration was about 7 µM and molar ratio [ANS]/[protein] was varied in the range 0.1 – 2.8. Dilutions on ANS never exceeded 5% of the starting volume. ANS and protein concentrations were spectrophotometrically determined. ANS emission spectrum was measured under excitation at λ_exc_ = 380 nm. During thermal aggregation induced by incubation at 70°C, time evolution of ANS fluorescence emission, was studied at [ANS]/[protein] molar ratio 0.3. Experimental errors for fluorescence and Rayleigh scattering measurements are estimated within 2% and they take into account for the instrument resolution, experiment repeatability including sample preparation, cuvette positioning and lamp stability. This value constitutes an upper limit.

### Data analysis

Intrinsic and ANS fluorescence emission spectra during the aggregation kinetics were analysed by calculating the moments of the spectral distribution. Zeroth (M_0_) and first (M_1_) moments were calculated according to the following definition [74]

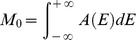


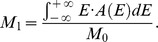
where *E* is the emission energy and *A(E)* is the spectral distribution obtained from the experimental data, after correcting for instrument response and subtracting the tangent to the minima of each band. M_0_ measures the integrated intensity and M_1_ is the mean value of the emission energy and measures the band position. This kind of analysis allows overcoming artifacts due to changes in shape of the emission band during the kinetics and allows interpretation of data without hypotheses on the origin of multiple components in the fluorescence signal.

### Circular dichroism

Measurements were performed using a Jasco J-715 spectropolarimeter, equipped with a Jasco PCT 348WI temperature controller. Complete CD spectra, as well as, single wavelength CD data during kinetics experiments were taken in the far UV region. Sample cell paths were 0.2 and 0.5 mm for complete spectra and single wavelength measurements, respectively. Spectra were sequentially recorded, using a scan speed of 50 nm/min, 1 nm bandwidth and a data pitch of 0.1 nm. For measurements at RT 10 spectra were averaged in order to improve signal to noise ratio. In single-wavelength measurements, no average of scans was employed. Variations in the absorption spectra and significant changes in the HT value during these measurements were not observed.

### FT-IR measurement

Single-beam IR spectra were measured using a Bruker (Ettlingen, Germany) Vertex 70 spectrophotometer with a spectral resolution of 2 cm^−1^, in the range 400–4000 cm^−1^ after purging the sample compartment with nitrogen to reduce water vapor. Each measurement is the result of the average of 200 scans. For Amide absorption, HSA and OX-HSA samples were dissolved in D_2_O at 15 mg/ml and placed between two CaF_2_ windows separated by a 0.025-mm Teflon spacer. D_2_O spectrum measured in the same conditions was subtracted after suitable normalisation. Vibrational peaks characteristics of methionine oxidation were observed in the 900– 1100 cm^−1^, measurements were carried out in solid HSA and OX-HSA KBr pellets at protein concentration of 0.5% w/w.

### TEM measurements

Aggregates were imaged according to a standard protocol [90], [91]. Copper 400 mesh grids (Agar Scientific, Stansted, UK) were coated with Formvar and carbon film. Aggregate solutions were diluted 50-fold in eppendorf tubes, and 5 µl aliquots were placed on the grids. After 60 s, 10 µl of distilled water was added and then excess water was removed. Then, 10 µl of 2% uranyl acetate (Agar Scientific) was placed on the grid and left for 30 s. Finally, two 10 µl drops of distilled water were added and again excess water removed. The grid was then left to dry. Images were collected using transmission electron microscopy (Technai 20, FEI) operating at an acceleration voltage of 120 kV and magnifications typically around ×26,000. It is worth to note that for high hierarchical assembly as amyloid aggregates the staining can be highly heterogeneous, mainly due to the compactness of the structures and salt exclusion. As a consequence, differences in the staining efficiency between different morphologies of aggregates can occur as also evidenced in Figure 8.

## Supporting Information

File S1 **Supporting figures.** Figure S1, Near UV CD spectra of HSA and OX-HSA. Figure S2, Intrinsic emission spectra of HSA and OX-HSA at different excitation wavelengths. Figure S3, Intrinsic excitation spectra of HSA and OX-HSA. Figure S4, Analysis of high concentration samples in the Amide I region. Figure S5, Thioflavin T emission spectra. Figure S6, SDS-PAGE gels of HSA and OX-HSA. Figure S7, Analysis of mass spectrum.
(PDF)
