# Supplementary material for: Oxidation Enhances Human Serum Albumin Thermal Stability and Changes the Routes of Amyloid Fibril Formation
Source: PLoS One. 2014 Jan 8;9(1):e84552. doi: 10.1371/journal.pone.0084552 (PMC3885593; doi:10.1371/journal.pone.0084552)
Supplement: File S1 — Supporting figures. Figure S1, Near UV CD spectra of HSA and OX-HSA. Figure S2, Intrinsic emission spectra of HSA and OX-HSA at different excitation wavelengths. Figure S3, Intrinsic excitation spectra of HSA and OX-HSA. Figure S4, Analysis of high concentration samples in the Amide I region. Figure S5, Thioflavin T emission spectra. Figure S6, SDS-PAGE gels of HSA and OX-HSA. Figure S7, Analysis of mass spectrum. (PDF) [file pone.0084552.s001.pdf]

Supplementary Materials

Oxidation Enhances Human Serum Albumin Thermal Stability and Changes the Routes of Amyloid Fibril Formation.

Giuseppe Sancataldo, Valeria Vetri, Vito Foderà, Gianluca Di Cara, Valeria Militello and Maurizio Leone.

Figure S1

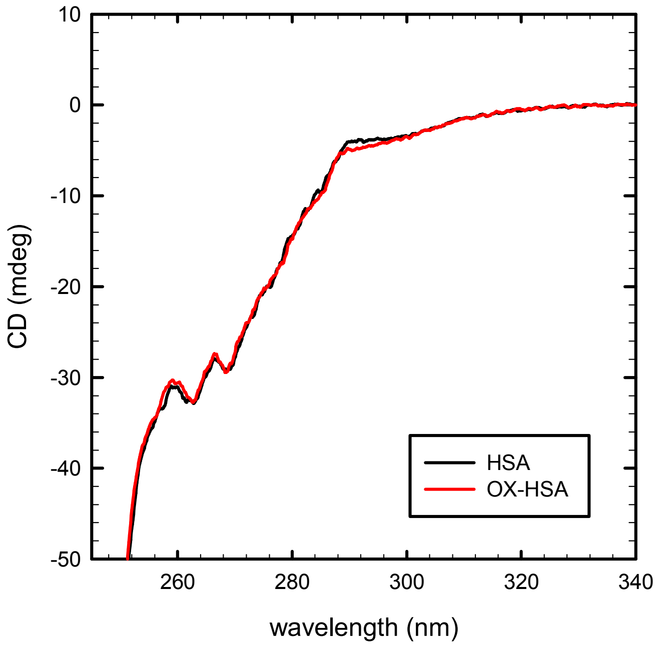

Figure S1. Near UV CD spectra of HSA (black) and OX-HSA (red). Spectra show two minima at 262 nm and 268 nm and a shoulder at 292 nm characteristic of aromatic chromophores and disulphide bonds. No significative variations are observed in native and oxidized sample suggesting no appreciable variation in the asymmetry of aromatic residues environment.

Figure S2

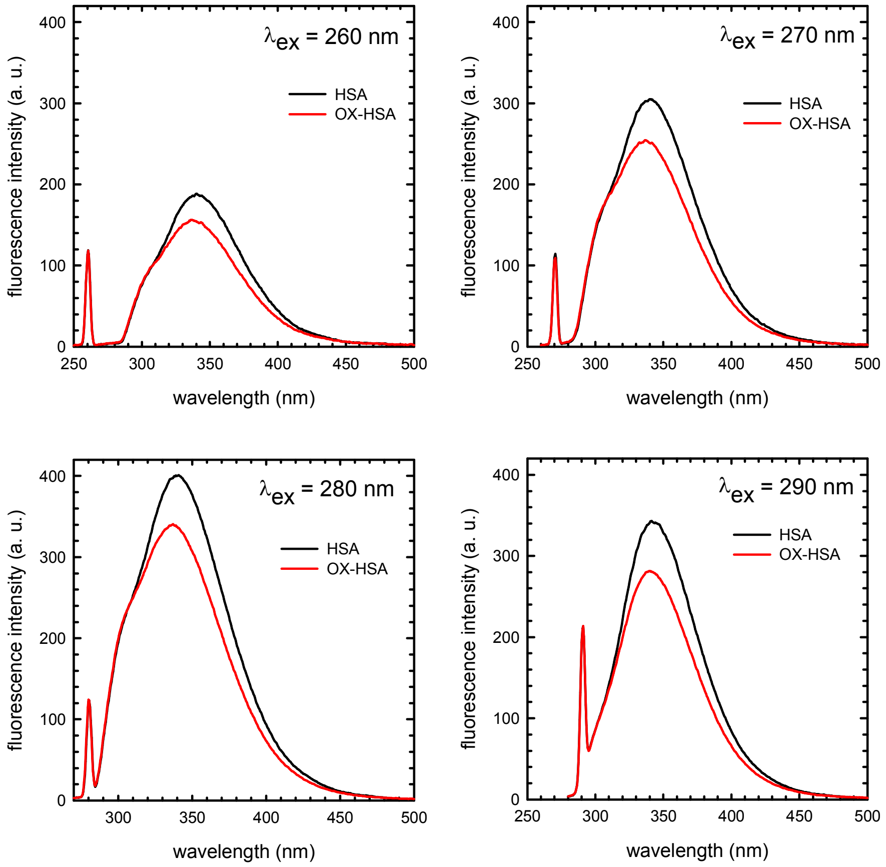

Figure S2. Intrinsic emission spectra of HSA (black) and OX-HSA (red) at different excitation wavelengths. Emission spectra, attributable to tyrosine and tryptophan, show a reduced quantum yield for OX-HSA sample. Emission spectrum excited at λ_{ex} = 290 nm does not show the shoulder observable in other spectra at about 310 nm due to the contribution of tyrosine, furthermore this spectrum shows a blue shift of the emission band in OX-HSA suggesting a more hydrophobic environment for tryptophan in oxidized sample.

Figure S3

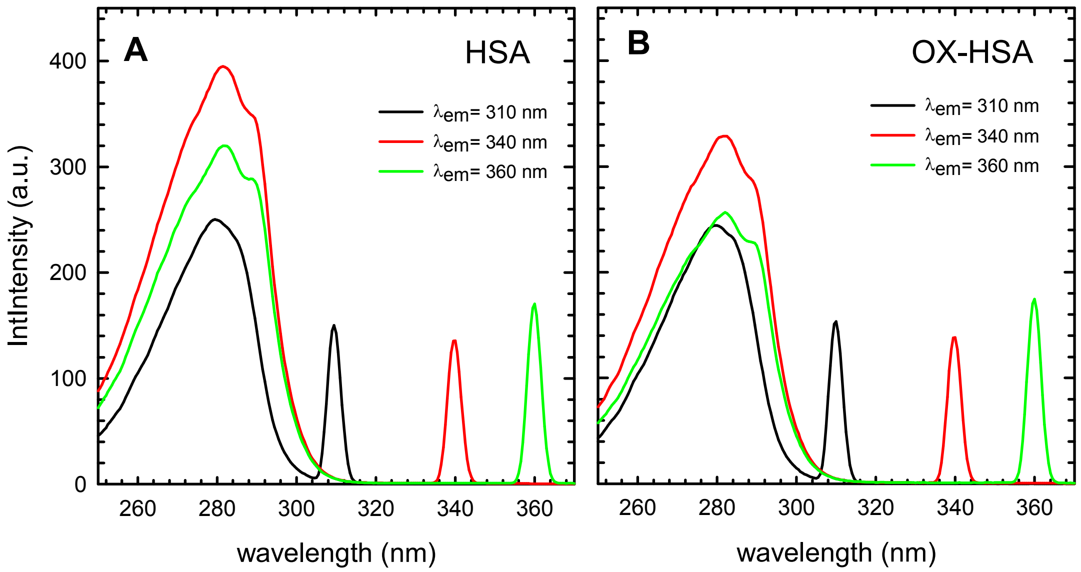

Figure S3. Intrinsic excitation spectra of HSA (A) and OX-HSA (B). In emission spectrum at λ_{em}=310 nm (black), where absorption contribution of tyrosines is prevalent, no significant variation is observed in OX-HSA and HSA sample. At longer wavelengths, where the contribution of the tryptophan is predominant, OX-HSA sample presents an excitation spectrum which, although similar in form, is characterized by a lower intensity compared to HSA sample. This is particularly evident in the excitation spectra measured atemission wavelength of λ_{em}=360 nm (green). This result suggests a significant variation in the tryptophan surrounding environment due to oxidation.

Figure S4

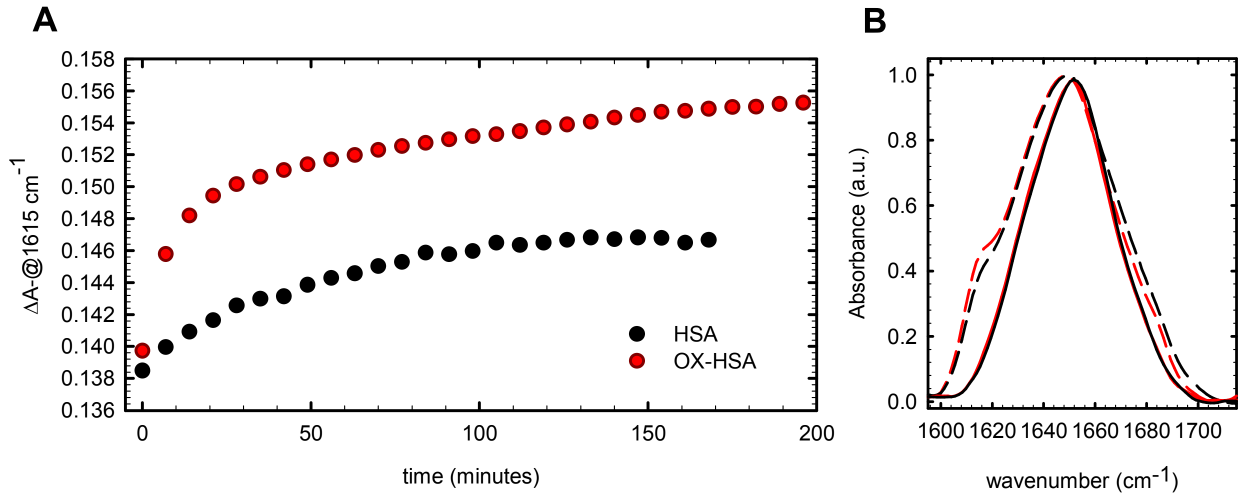

Figure S4. Analysis of high concentration samples in the Amide I region. (**A**) Temporal evolution of absorbance at 1615 cm^{-1} during aggregation Kineticsat 70 °C of 10 mg/ml HSA (black dots) and OX-HSA (red dots). (**B**) Normalized absorption spectra of freshly prepared samples HSA (black solid line), OX-HSA (red solid line) and of samples after incubation at 70°C for 200 min HSA aggregates (black dashed line) and OX-HSA aggregates (red dashed line). As can be seen the temporal evolution of absorbance at 1615 cm^{-1} is different being variations in OX-HSA sample of larger extent. The shape of aggregates samples presents the typical peak around 1615 cm^{-1} of parallel intermolecular β-sheets and shoulder at 1680 cm^{-1}assigned to antiparallel β-sheets. The ratio between these signals is different in the two samples clearly indicating that the two aggregates are different at secondary structure level. These measurements are in line with what found at lower concentration in CD experiments.

Figure S5

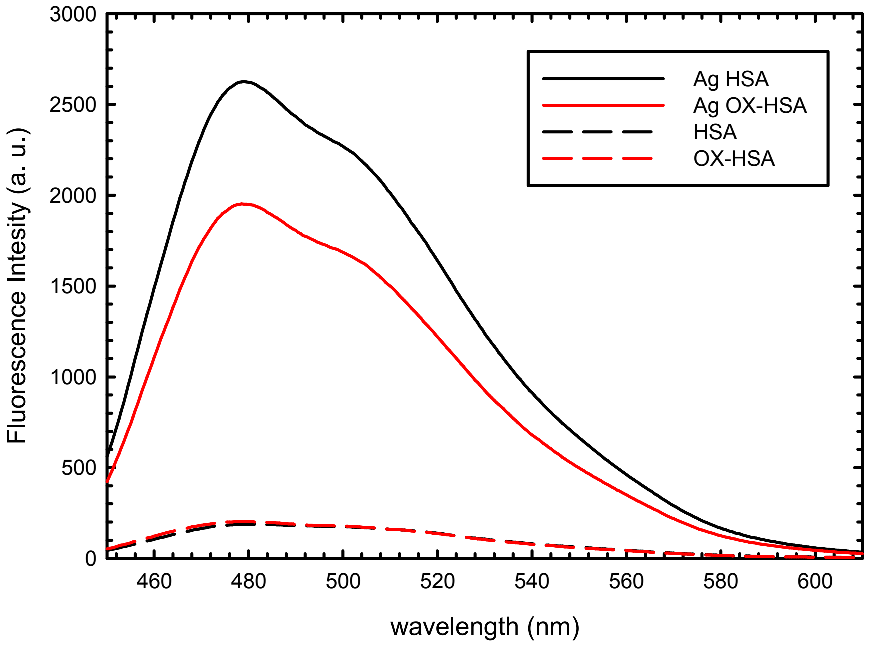

Figure S5. Thioflavin T emission spectra. Room Temperature *ex situ* ThT Emission spectra of native HSA (dashed black line), OX-HSA (dashed red line), HSA aggregates (solid black line) and OX-HSA aggregates (solid red line). Measurements were performed in protein 0.5 mg/ml (7 μM) and ThT40 μM concentration in K-phosphate buffer at pH 7.4 for freshly prepared sample and sample after incubation at 70°C for 12h. OX-HSA sample shows a lower Tht intensity suggesting a less amyloid-like nature for the OX-HSA aggregates.

Mass spectroscopy analysis

HSA and OX-HSA were compared by means mass spectroscopy measurements (performed according to the protocol previously described in *Di Cara et. al Anticancer Res. 2013 Feb;33(2):489-503).*

As can be seen in Figure S7 electrophoresis measurements on HSA sample revealed a unique major band (lane **b**) corresponding to molecular weight of monomeric HSA. In OX-HSA sample a broadening of the band at this molecular weight is observed highlighting the presence of higher molecular weight components in the sample. In particular, it is possible to roughly distinguish two main lanes: **a** and **c**. The different migration behavior clearly indicates that some modification occurred in OX-HSA due to H_2O_2 treatment.

Within the experimental error, molecular weight in OX-HSA sample appears to be distributed over a maximum of 2500 Da higher than HSA monomer molecular weight, this fraction is much less than 20% of the sample. MALDI spectra of samples in **a**, **b** and **c** were performed and peptide mass fingerprinting was compared to the theoretical masses from the Swiss-Prot or U.S. National Center for Biotechnology Information (NCBI, Rockville Pike, Bethesda, MD, USA) sequence databases using Mascot (http://www.matrixscience.com/) The analysis of MALDI spectra of protein fraction in lane **c** (i.e. where larger modification occurred) reveals Cys and Met oxidation in 3 peptides and clearly indicates that the unique tryptophan present in the HSA polypeptide chain is not oxidized.

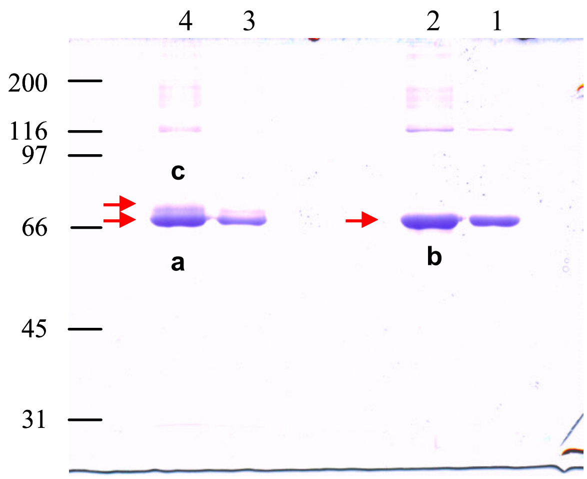

Figure S6. SDS-PAGE gels of HSA (1 and 2) and OX-HSA (3 and 4) samples. The molecular weight reference standard (BioRad, 31 – 200 kDa scale) are marked on the left.

1. ALBU_HUMAN Mass: 69321 Score: 67 Expect: 0.0039 Matches: 21

Serum albumin OS=Homo sapiens GN=ALB PE=1 SV=2

Observed	Mr(expt)	Mr(calc)	ppm	Start	End	Miss	Peptide
673.3720	672.3647	672.3707	-8.93	237 -	242	0	K.AWAVAR.L
695.3554	694.3481	694.3286	28.2	342 -	347	0	K.NYAEAK.D
875.4234	874.4161	874.5025	-98.74	243 -	249	1	R.LSQRFPK.A
880.4113	879.4040	879.4338	-33.83	250 -	257	0	K.AEFAEVSK.L
927.4882	926.4809	926.4861	-5.62	162 -	168	0	K.YLYEIAR.R
940.4647	939.4574	939.4410	17.5	131 -	138	0	K.DDNPNLPR.L
951.4629	950.4556	950.4345	22.2	37 -	44	0	K.DLGEENFK.A
960.5775	959.5702	959.5552	15.6	427 -	434	0	K.FQNALLVR.Y
1000.5467	999.5394	999.5964	-57.04	550 -	558	0	K.QTALVELVK.H
1017.5261	1016.5188	1016.5291	-10.11	89 -	97	0	K.SLHTLFGDK.L
1074.5659	1073.5586	1073.5353	21.7	206 -	214	1	K.LDELRDEGK.A
1149.5852	1148.5779	1148.5686	8.08	25 -	34	1	R.DAHKSEVAHR.F
1226.6081	1225.6008	1225.5979	2.40	35 -	44	1	R.FKDLGEENFK.A
1352.6693	1351.6620	1351.6588	2.39	497 -	508	1	R.VTKCCTESLVNR.R
1384.6692	1383.6619	1383.6486	9.60	497 -	508	1	R.VTKCCTESLVNR.R + 2 Oxidation (C)
1467.8582	1466.8509	1466.8358	10.3	361 -	372	1	R.RHPDYSVVLLLR.L
1639.9223	1638.9150	1638.9305	-9.43	438 -	452	1	K.KVPQVSTPTLVEVSR.N
1708.7921	1707.7848	1707.7307	31.7	570 -	584	1	K.AVMDDFAAFVEKCCK.A + 2 Oxidation (C)
1853.9049	1852.8976	1852.9029	-2.86	509 -	524	0	R.RPCFSALEVDETYVPK.E
2045.0875	2044.0802	2044.0881	-3.84	397 -	413	0	K.VFDEFKPLVEEPQNLIK.Q
2875.4232	2874.4159	2874.3350	28.1	500 -	524	1	K.CCTESLVNRRPCFSALEVDETYVPK.E + Oxidation (C)

Figure S7. Results of analysis of mass spectrum in bands c. Note that Methionines oxidation can not be evaluated from this result.
